# Nutrition interventions to address nutritional problems in HIV-positive patients: translating knowledge into practice

**DOI:** 10.1186/s41043-023-00440-z

**Published:** 2023-09-08

**Authors:** Leila Rezazadeh, Alireza Ostadrahimi, Helda Tutunchi, Mohammad Naemi Kermanshahi, Samira Pourmoradian

**Affiliations:** 1 Nutrition Research Center, Faculty of Nutrition and Food Sciences, Tabriz University of Medical Sciences, Tabriz, Iran; 2 Nutrition Research Center, Department of Clinical Nutrition, Tabriz University of Medical Sciences, Tabriz, Iran; 3 Endocrine Research Center, Tabriz University of Medical Sciences, Tabriz, Iran; 4 Student Research Committee, Nutrition Research Center, Faculty of Nutrition and Food Sciences, Tabriz University of Medical Sciences, Tabriz, Iran; 5 Nutrition Research Center, Department of Community Nutrition, Faculty of Nutrition and Food Sciences, Tabriz University of Medical Sciences, Tabriz, Iran

**Keywords:** HIV, NACS, Nutrients, Malnutrition, Nutrition counseling, Nutrition interventions

## Abstract

**Background:**

Human immunodeficiency virus (HIV) infection and malnutrition negatively reinforce each other. Malnutrition leads to further immune deficiency and accelerates disease progression. The present overview aimed to investigate the current knowledge from review articles on the role of nutrition interventions as well as food and nutrition policies on HIV-related outcomes in adults to present future strategies for strengthening food and nutrition response to HIV.

**Methods:**

We searched PubMed/Medline, Scopus, Embase, ProQuest, and Ovid databases using the relevant keywords. The search was limited to studies published in English until April 2022. All types of reviews studies (systematic review, narrative review, and other types of review studies) which evaluated the impact of nutritional program/interventions on HIV progression were included.

**Results:**

Although nutrition programs in HIV care have resulted in improvements in nutritional symptoms and increase the quality life of HIV patients, these programs should evaluate the nutritional health of HIV-infected patients in a way that can be sustainable in the long term. In additions, demographic, clinical, and nutritional, social characteristics influence nutritional outcomes, which provide potential opportunities for future research.

**Conclusion:**

Nutrition assessment, education and counseling, and food supplements where necessary should be an integral part of HIV treatment programs.

## Background

Human immunodeficiency virus (HIV) is a serious communicable disease characterized by immunodeficiency and other complications that increase mortality rate in these patients. In 2020, it was estimated that the people living with HIV were 37.7 million worldwide, and 1.5 million people became newly infected with HIV [1]. According to the World Health Organization, HIV-related deaths were estimated about 630,000 cases by 2022. The prevalence of malnutrition (undernutrition) in HIV patients in some African countries is over 25% [2].

HIV is known by immune system suppression which increases energy requirement to combat infection in undernourished HIV patients, leading to further nutritional problems [3, 4]. Malnutrition is an important health issue in patients living with HIV. Malnutrition leads to physiological, psychological, and functional disorders. In addition, malnutrition contributes to further immune deficiency and accelerates disease progression. The underlying causes of malnutrition in HIV-infected person are related to reduced food intake, poor absorption, changes in metabolism, chronic infections, and illnesses [5]. Adequate nutrition, which provides sufficient calories by macronutrients (proteins, carbohydrates and fats) and micronutrients (vitamins and minerals), is important to increase resistance to infection, maintain nutritional status of patients, help to delay the progression of HIV, and improve quality of life [4, 6, 7].

In developing countries, poor nutrition status and food insecurity increase individuals’ susceptibility to infectious diseases, as well as viral load, sexual, and vertical transmission of HIV [8]. Nutrition interventions including supplementation with macro- and micronutrients, nutrition education, or counseling and food assistance programs are essential for HIV-positive persons to improve nutrient intake and reduce viral load by enhancing immunity [9, 10, 11].

To date, several review and systematic review studies have evaluated the effectiveness of such interventions on nutritional status of HIV. Considering sociodemographic, clinical and nutritional characteristics of various populations can influence the nutritional outcomes that provide potential opportunities for improvement in future research and programs. As far as we know, there is no overview to summarize the results of these review studies. Therefore, the present overview aimed to investigate the current knowledge from review articles on the role of nutrition interventions as well as food and nutrition policies on HIV-related outcomes in adults to present future strategies for strengthening food and nutrition response to HIV.

## Material and methods

For the purposes of this overview, we searched PubMed/Medline, Scopus, Embase, ProQuest, and Ovid from 2000/01/01 until 2022/04/30. We developed and performed the literature search (MN, LR).

Only publications with English language were included.

Search strategy included combinations of the following terms:

“HIV” OR “human immunodeficiency virus*” OR “HIV” OR “People Living with HIV” OR “PLWHIV” OR “PLHIV” OR “HIV-positive” OR” HIV positive” AND “Nutrition program” OR “Nutritional intervention” OR “Nutrition education” OR “Micronutrient supplementation” OR “Macronutrient supplementation” OR “Ready to use Therapeutic food” OR “complementary therapy” OR “food assistance program”.

Gray literature (e.g., conference papers, theses, interviews, protocols, comments, and short communications) was obtained through Google searches and Elsevier (first 20 pages of results). Apart from electronic search, manual search of the reference lists of the eligible papers and relevant review articles were conducted to avoid missing relevant studies.

### Inclusion and exclusion criteria

Two of the authors (HT and SP) screened the title and abstract of all studies found in the systematic search to identify studies that met our criteria for inclusion in the present study. Systematic review which evaluated the impact of nutritional program/interventions on HIV progression was included. However, studies which examined nutrition intervention on children with HIV and also pregnant and lactation women were excluded.

### Quality assessment of included reviews

Two authors (OA and LR) assessed the quality of included SRs using A Measurement Tool to Checklist Assess Systematic Reviews 2 (AMSTAR 2). AMSTAR2 is a practical appraisal tool for SRs performed on randomized and/or nonrandomized studies of healthcare interventions. It contains16 domains. Each question was answered with “yes,” “no,” “cannot answer,” or “not applicable.” According to the answers, only the “yes” answer counted as a point in the total score for the assessed study. Thus, the meta-analyses with at least 80% of the items were categorized as high quality and those SRs between 40 and 80% scores were considered as moderate quality, and those with < 40% of the items were satisfied as low quality [12].

## Results

### Characteristics of included studies

**Fig. 1:**
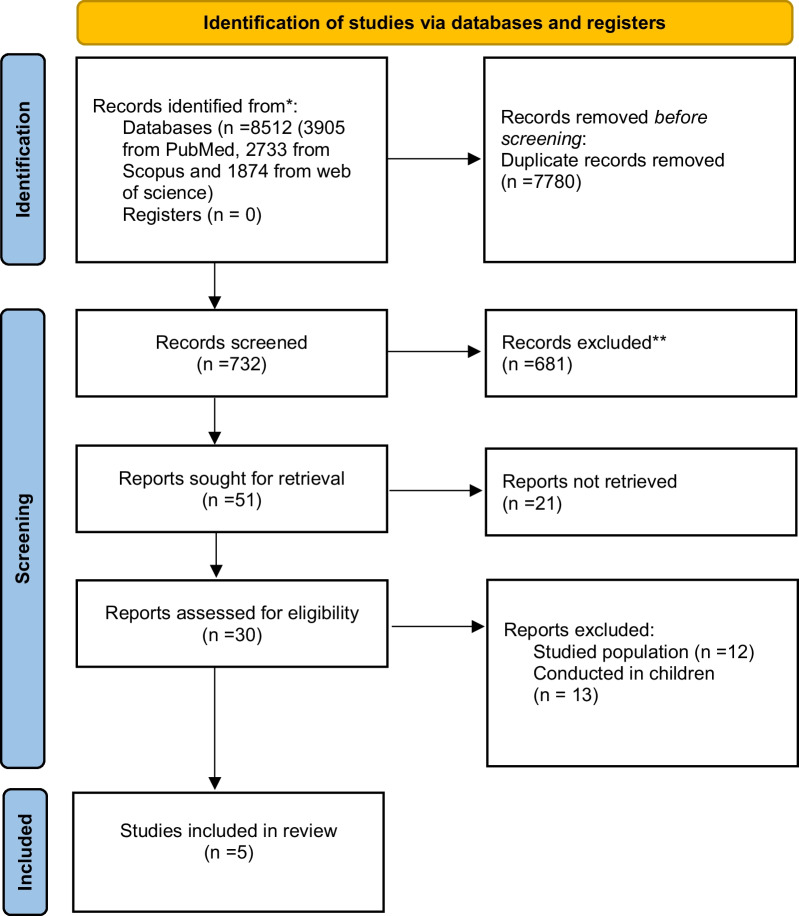
The PRISMA flowchart of the study

After excluding duplicates and irrelevant studies based on screening of the title and abstract, 30 full-text articles were reviewed in detail for eligibility and finally 5 studies met our specified inclusion criteria. The PRISMA flowchart of the study is shown in Fig. 1.

Table 1 shows the characteristics of the five included SRs of the effects of nutritional program/interventions in HIV-positive adults [10, 13–16]. The nutrition interventions for HIV patients are classified into three groups including education/counseling, supplementation with macro- and micronutrients, and food and nutrition supporting programs. Two of five included SRs were Cochrane systematic reviews [13, 15]. Most of the included studies were searched at least two databases including Embase and PubMed/Medline.

**Table 1:** The characteristics of the included SRs of the effects of nutritional program/interventions in HIV-positive adults

Author/date	Policy type	Type of the study	Policy description	Primary outcome	Findings
Hong et al. [14]	Macronutrient supplementation	Systematic review and meta-analysis	Evaluation of the effects of protein-energy-fortified macronutrient supplements	Weight, BMI, fat-free mass, and CD4 count	A significant change in nutritional status and immunological response
Grobler et al. [13]	Macronutrient interventions fortified with micronutrients and nutrition counseling	Cochrane (systematic reviews)	Evaluation the effectiveness of various macronutrient interventions such as amino acids, whey protein concentration or spirulina in reducing morbidity and mortality	Weight, BMI, fat-free mass, fat mass, viral load, CD4 count, and dietary intake	A significant improvement in energy (increased energy intake by 394 kcal/day in the intervention arm) and protein intakes (increased daily protein intake by 23.25 g/day compared with no supplements) compared with no nutritional supplementation or nutrition counseling alone in adult participants with weight loss, after supplementation with macronutrient formulas providing protein and/or energy and fortified with micronutrients, in conjunction with nutrition counseling
Tesfay et al. [10]	Nutritional assessment, counseling and nutritional support	Systematic review	Impacts of nutritional assessment, counseling and nutritional support to address undernutrition	BMI, weight, and MUAC	Short-term improvements in weight-related nutritional outcomes
Mahlungulu et al. [15]	macronutrient supplementation and nutrition counseling	Cochrane (systematic reviews)	Evaluation the effectiveness of various macronutrient interventions, such as a balanced diet or high protein, high carbohydrate, or high fat diets given orally	Weight, BMI, fat-free mass, fat mass, and CD4 count	Significant improvement in energy (367 kcal day) and protein intakes (17 g day) and no evidence of an effect on body weight, fat mass, fat-free mass or CD4 count with macronutrient supplementation (with or without nutritional counseling)
Tang et al. [16]	Nutrition assessment, counseling, and support (NACS) interventions	Systematic review	Examination of data on nutrition assessment, counseling, and support (NACS) interventions on mortality, morbidity, retention in care, quality of life, and/or prevention of ongoing HIV transmission	Mortality, morbidity, retention in care, quality of life, and ongoing HIV transmission	Improvement in both short- and long-term patient retention in care and treatment, and clinical outcomes

*HIV* human immunodeficiency virus, *CD4* cluster of differentiation 4, *CAM* complementary and alternative medicine, *NACS* nutrition assessment, counseling, and support, *PIs* protease inhibitors, *HAART* highly active antiretroviral therapy treatment

The three of included SRs were assessed the macronutrient supplementation in HIV-positive adults [13–15]. Two studies conducted by Tesfay et al. [10] and Tong et al. [16] were evaluated nutrition assessment, counseling, and support (NACS) interventions.

The primary outcomes of included studies were weight, BMI, fat-free mass, fat mass, viral load, CD4 count, and dietary intake.

The intervention location in the most of the included SRs was outpatients clinics or community-based settings [13–15]. Furthermore, in the two SRs, the multiple setting interventions (conducted in the hospital and outpatient clinic or community-based settings) were included [10, 16].

### Methodological quality of the included reviews

The methodological quality of included SRs articles is depicted in Table 2. According to AMSTAR2 quality assessment tool, SRs conducted by Grobler et al. [13], Mahlungulu et al. [15] which were Cochrane systematic reviews had high quality. The Hong et al. [14] and Tang et al. [16] studies had moderate quality, and only one of them possess low quality [10] (Table 2).

**Table 2:** A measurement tool to checklist assess systematic reviews 2 (AMSTAR2) assessment for each systematic review

AMSTAR items																	
References	1	2	3	4	5	6	7	8	9	10	11	12	13	14	15	16	Score (%)
Hong et al. [14]	Yes	No	Yes	Yes	No	No	Yes	Yes	Yes	No	Yes	Yes	Yes	Yes	Yes	Yes	75% (moderate)
Grobler et al. [13]	Yes	Yes	Yes	Yes	Yes	Yes	Yes	Yes	Yes	Yes	Yes	Yes	Yes	Yes	Yes	Yes	100% (high)
Tesfay et al. [10]	Yes	No	Yes	No	No	No	Yes	No	Yes	No	Cannot answer	Cannot answer	No	Yes	Cannot answer	Yes	37.5% (low)
Mahlungulu et al. [15]	Yes	Yes	Yes	Yes	Yes	Yes	Yes	Yes	Yes	Yes	Yes	Yes	Yes	Yes	Yes	Yes	100% (high)
Tang et al. [16]	Yes	No	Yes	Yes	Yes	Yes	Yes	Yes	Yes	No	Cannot answer	Cannot answer	No	Yes	Cannot answer	Yes	62.5% (moderate)

## Discussion

Nutritional intervention plays a crucial role in the management of HIV infection. People living with HIV often experience malnutrition and weight loss due to various factors such as decreased appetite, nutrient malabsorption, increased energy expenditure, and opportunistic infections [17]. Nutritional intervention is a critical component of the overall management of HIV infection. It helps support immune function, prevent malnutrition, and improve overall health outcomes for individuals living with HIV. Here, we discuss our findings in three sections.

### Nutrition education/counseling

Nutrition education or counseling has been shown to assist and improve dietary intake and nutritional status in HIV-positive adults in different settings by increasing individual knowledge about healthy food choices [10, 13–16]. In studies conducted by Grobler et al. [13], Mahlungulu et al. [15], nutrition education or counseling was one component of the nutritional programs in HIV-positive patients.

Despite the value of nutrition education/counseling in HIV treatment programs, there is little research in this field. Nutrition counseling as a cost-effective intervention especially in developing countries with limited resources and high prevalence of nutritional deficiencies must be in the first line of nutrition intervention in HIV management [8]. To improve the health and nutrition status of people living with HIV, the expertise of a registered dietitian/nutritionist as part of the health care team should be considered.

### Supplementation with macro- and micronutrients

Based on previous evidences, reduction of serum antioxidant vitamins and minerals was reported during the progression of HIV with high oxidative stress. So, intervention with foods and supplements including macro- and micronutrients is necessary for strengthen the immune system. Furthermore, people with HIV should be aware of health benefits of essential nutrients along with lifestyle changes to improve nutritional status and the immune system [18]. The effectiveness of supplementation with macro- and micronutrients was reviewed by Hong et al. to improve immune defense system and quality of life of HIV patients. They found that supplementation with selenium, zinc, iron, probiotic, vitamins A, B, C, and E improved quality of life and immunity in HIV-infected patients. Furthermore, Hong et al. [14] showed that protein-energy-fortified macronutrient supplements led to significant changes in nutritional status and immunological response in HIV disease.

Given the potential effectiveness of the supplementation of macronutrients and micronutrients in HIV, it is necessary to establish national guidelines for health care provider as well as evaluating socio-psychophysiological status to assist in their clinical decision in terms of improving nutritional status and immunologic responses [14].

### Multiple interventions

Adequate nutrition is recognized as a key component in the care and support for people living with HIV due to the high prevalence of clinical undernutrition and food insecurity. There is a strong association between malnutrition and increased mortality rate in HIV-infected patients [19].

According to the nutrition requirement guideline of the World Health Organization (WHO), providing a balanced healthy diet as well as increasing energy intakes by approximately 20% to 30% to maintain body weight is vital for health and survival in individuals with HIV [20].

Nutrition assessment, counseling, and support (NACS) interventions were designed for evaluating nutritional status of undernourished people. NACS includes a wide range of interventions to prevent and treat malnutrition among HIV-infected adolescents and adults in clinical care in low-resource settings [16, 21].

There were two review studies that examined the effect of NACS interventions on mortality, morbidity, retention in care, quality of life, prevention of HIV transmission, and weight-related nutritional outcomes [10, 16]. In the systematic review conducted by Tang et al. [16], NACS interventions had no significant impact on all five outcomes (mortality, morbidity, retention in care, quality of life, and prevention of ongoing HIV transmission) in HIV-infected patients. Tesfay et al. showed that nutritional programs in HIV care led to some improvements in nutritional outcomes related to body weight in HIV people. However, the long-term nutrition status indicators such as food security were recommended to assess the nutritional status of people living with HIV [10]. In three narrative review studies, the multicomponent nutrition interventions including nutritional education, food aid, supplementation of macro- and micronutrients, exercise, and livelihood interventions were assessed on nutritional outcomes, quality of life, and mortality in HIV patients [9, 20, 22]. The available evidence suggests that nutrition education is an essential component in all settings accompanied by food assistance program as well as supplementation of macro- and micronutrients in resource-limited settings [20]. However, they reported an increase in quality of life and a decrease in mortality, as well as improvements in metabolic abnormalities and body composition following multivitamin supplementation, nutritional counseling, and exercise interventions in HIV-infected individuals [9, 22].

## Strengths and limitations

The key strength of this study is its coverage of all type of interventions in this field as well as summarizing the results of various nutrition programs in HIV patients in SRs studies. It also examines a range of HIV symptoms. However, the review has some limitations. This study only included articles published in English that may have omitted papers on a same topic that have been published in other languages. Furthermore, all reviewed studies in present review evaluated the impact of nutritional programs on undernourished individuals living with HIV in health care settings, while overnutrition as a risk factor of overweight and cardiovascular disease is important in these people and therefore this issue should be considered for future studies.

## Conclusion

The findings of this review indicate that nutritional interventions in HIV care lead to improved nutritional symptoms and increased quality of life in these patients. However, nutritional programs on HIV care should evaluate the nutritional health of HIV-infected patients in a way that can be sustainable in the long term. In additions, demographic, clinical, nutritional, and social characteristics influence nutritional outcomes, which provide potential opportunities for future research.

## Data Availability

Not applicable.
